# The impact of perioperative adverse events on kidney transplant surgeons: a mixed-methods study

**DOI:** 10.3389/frtra.2026.1959604

**Published:** 2026-10-14

**Authors:** Dominic Laverty, Karen Stevenson, Catrin Jones, Carly Bisset, Susan Moug, Emma Aitken

**Affiliations:** 1 Medical School, University of Glasgow, Glasgow, United Kingdom; 2 Department of Renal Transplantation, Queen Elizabeth University Hospital, Glasgow, United Kingdom; 3 Department of General Surgery, Golden Jubilee National Hospital, Clydebank, United Kingdom

**Keywords:** adverse outcomes, personality, psychology, renal transplant, surgeon skill

## Abstract

**Aim:**

Adverse events (AEs) are an inevitable part of every surgeon's career. There is currently no evidence regarding the impact of perioperative AEs on transplant surgeons. This study aims to evaluate the impact of perioperative AEs on kidney transplant surgeons, exploring the emotional, professional and personal consequences when they occur.

**Method:**

A mixed-methods exploratory sequential design was used. Purposive sampling was completed to recruit 15 consultant kidney transplant surgeons to be interviewed about an AE that they had experienced. Individual, semi-structured qualitative interviews were transcribed verbatim and analysed. Alongside this, participants completed two personality questionnaires: the Big Five Inventory and the DOSPERT attitude to risk questionnaire. Integration of the two data sets occurred during the analysis stage.

**Results:**

Four main themes with relevant subthemes were identified: (1) AEs have a psychological impact; (2) the surgical environment influences response; (3) changes in practice following an AE; (4) the influence of personality on response. Triangulation of findings displayed how external factors such as personality, severity of the AE, seniority and the family's reaction can all influence how AEs are processed.

**Conclusion:**

This study highlights the psychological and professional impact of perioperative AEs on kidney transplant surgeons. Findings suggest team dynamics, coping strategies and identity shape responses. Further research is needed to inform future support services that are currently not routinely relied upon by transplant surgeons.

## Introduction

An adverse event (AE) is “a harmful and negative outcome that happens when a patient has been provided with medical care” (1). AEs are associated with patient harm and increased length of hospital stays (2). Notably, most in-hospital AEs are related to surgical care, with approximately 14.4% of surgical patients experiencing an AE, and potentially preventable AEs occurring in 5.2% of cases (3).

The ‘Second Victim’ of an AE was a term coined by Albert Wu in 2000 to describe harm to the clinician incurred by an AE (4). This encompasses feelings of guilt, fear and anger, as well as concerns surrounding professional reputation, job satisfaction and confidence in their practice following a medical error. Together these personal and professional implications have been termed the ‘Second Victim Syndrome (SVS) (5).’

The existing evidence demonstrates the emergence of several common themes, including increased anxiety about future errors, loss of confidence and reduced job satisfaction (6–8). The extent of this response can be determined by the severity of the error (7, 8) - and the clinical context in which it occurs – with surgeons in particular facing distinct professional and clinical pressures.

There is an increased chance of severe AEs occurring within surgical specialties, and the scrutiny of these is heightened (9). Surgeons may experience greater feelings of accountability, where patient outcomes are seen to directly reflect their technical skills and decision-making (10, 11). A recent systematic review published in the *British Journal of Surgery,* co-authored by two members of the present study team, found that over half of surgeons experienced anxiety, guilt and sleep disturbance following an AE (8). Qualitative analysis of data from colorectal surgeons following anastomotic leak further highlighted the psychological burden of AEs, with surgeons describing changes in clinical practice and emotional distress (12, 13). Similar responses to AEs have also been reported among vascular (14), paediatric (15), general (16) and obstetrics and gynaecological surgeons (17). The psychological burden experienced across surgical specialties suggests surgeons may be at increased risk of developing SVS compared with other healthcare professionals (18, 19).

There is limited generalisability of these results to transplantation as it represents a uniquely ‘high stakes’ surgical field involving two patients – donor and recipient. With this comes the added responsibility of maintaining the safety of two patients whilst ensuring the organ doesn't go to waste. Outcomes are closely monitored with limited opportunity to rectify errors once the operation has been performed (20, 21). In addition, a surgeon's influence extends beyond the procedure itself, encompassing organ acceptance decisions on behalf of their patients. A shift toward risk aversion following AEs may lead to higher rates of organ refusals, and fewer life-saving transplants performed (22). There are currently no studies evaluating the effect of AEs and SVS in transplant surgery, which is notable given the likely additional medical, ethical and personal implications of AEs within transplantation.

This study aims to improve the understanding of the impact that AEs have on kidney transplant surgeons. It examines the physical, psychological, and emotional effects of these events, alongside their influence on clinical practice and perioperative risk-taking. The study aims to consider if surgeon personality shapes responses to AEs and future risk-taking behaviour, as well as to examine the current support available to kidney transplant surgeons experiencing AEs.

## Methodology

### Ethical approvals

Ethical approval was provided by the North-West England Research Ethics Committee, (23/NW/0374). All participants provided written informed consent. Research was carried out in line with the Declaration of Helsinki. Reporting uses the COREQ 32-item checklist as a framework (23).

### Study design

This mixed-methods study used an exploratory sequential design with semi-structured individual interviews, followed by quantitative data collection.

### Participant selection

UK-based consultant kidney transplant surgeons were recruited using a stratified purposive sampling technique. Email invitations were distributed by researchers to selected kidney transplant surgeons from seven units across the UK, ensuring a diversity of experience, gender and age. Eligibility criteria required participants to have experienced a perioperative AE during their tenure as a consultant in kidney transplantation. A perioperative AE was defined as an unintended event occuring during or following transplantation that resulted in patient harm. This encompasses errors (preventable mistakes that occur during an operation (24) and complications (detrimental outcomes that are known to be a risk of a procedure (25). For recruitment purposes, errors and complications were not used as separate eligibility criteria, allowing a broad range of perioperative AEs to be explored (Supplementary Table S1).

## Data collection

### Participant interviews

Interviews were conducted between March and December 2025 with the aid of a topic guide (Supplementary Appendix). The topic guide was developed to permit exploration of themes identified from existing relevant literature (12, 13, 18); reviewed by fellow researchers and piloted on two transplant surgeons, allowing refinement in clarity and wording.

The interviewer (D.L.), a male 4th year medical student, had minimal prior experience of kidney transplantation, and no prior engagement with participants who had been informed of his role. He had completed Trauma Informed Counselling Training (Open University, DGXS004) prior to undertaking the interviews. Only D.L. and the surgeon were present during the interview.

Data was collected through audio recording either in-person via secure Dictaphone or over Microsoft Teams (participant preference and geographical location determined). Interviews lasted between 22 and 71 min. No repeat interviews were conducted and no field notes taken. Interviews were transcribed verbatim and subsequently redacted by the interviewer to maintain anonymity. No transcripts were returned to participants, and they did not provide feedback on study findings.

### Participant questionnaires

Following the interviews, participants were asked to complete two short questionnaires: the Big Five Inventory (BFI) to assess personality and the Domain-Specific Risk-Taking (DOSPERT) scale attitude to risk score.

The BFI is an established 44-item questionnaire formed around the Five Factor model of personality (extraversion, agreeableness, conscientiousness, neuroticism and openness) (26, 27). Responses are ranked on a five-point Likert scale, 1 (disagree strongly) to 5 (agree strongly). Negatively keyed items are reverse-scored, and mean scores were calculated for each domain for each participant.

The DOSPERT Scale (Supplementary Appendix) provides insight into conventional risk attitudes (reported level of risk taking) and perceived-risk attitudes (the “assessment of riskiness of these behaviours”) (28). These are split into five broad domains: ethical, financial, health/safety, social and recreational. A seven-point scale (1 is extremely unlikely, 7 is extremely likely) is used to quantify the likelihood that respondents will engage in risk taking behaviour.

### Data analysis

Reflexive thematic analysis of qualitative data was performed according to Braun and Clarke's six-step framework allowing for both flexibility and deep engagement with the data (29). Coding and analysis were led by D.L., with 25% of codes independently reviewed by another researcher (C.J.). Initial coding was performed using NVivo software. Thereafter an iterative thematic structure of codes was discussed within the research team until consensus was reached. Interviews were continued until no new semantic codes were being generated.

Quantitative analysis of questionnaires was performed using Microsoft Excel and R studio. Mean scores from our cohort were compared with findings from other relevant studies (30, 31), to explore within-group variability.

Integration of the two data collection techniques was completed at the analysis stage. Qualitative coding alongside the quantitative data was visualised and discussed between two researchers (D.L., K.S.) who had reviewed both data sets.

Due to the limited sample size, quantitative data are reported using descriptive statistics at a domain level, and not analysed for explanatory or inferential purposes.

## Results

25 surgeons from seven UK transplant centres were approached. 15 consented to participate (nine non-responders, one incomplete interview).

Two participants were interviewed but did not complete questionnaires, giving a sample size of 13 for integrative analysis (Supplementary Figure S1). Participant demographics are reported in Supplementary Table S1.

Thematic analysis identified four key themes and nine subthemes (Figure 1).

**Figure 1:**
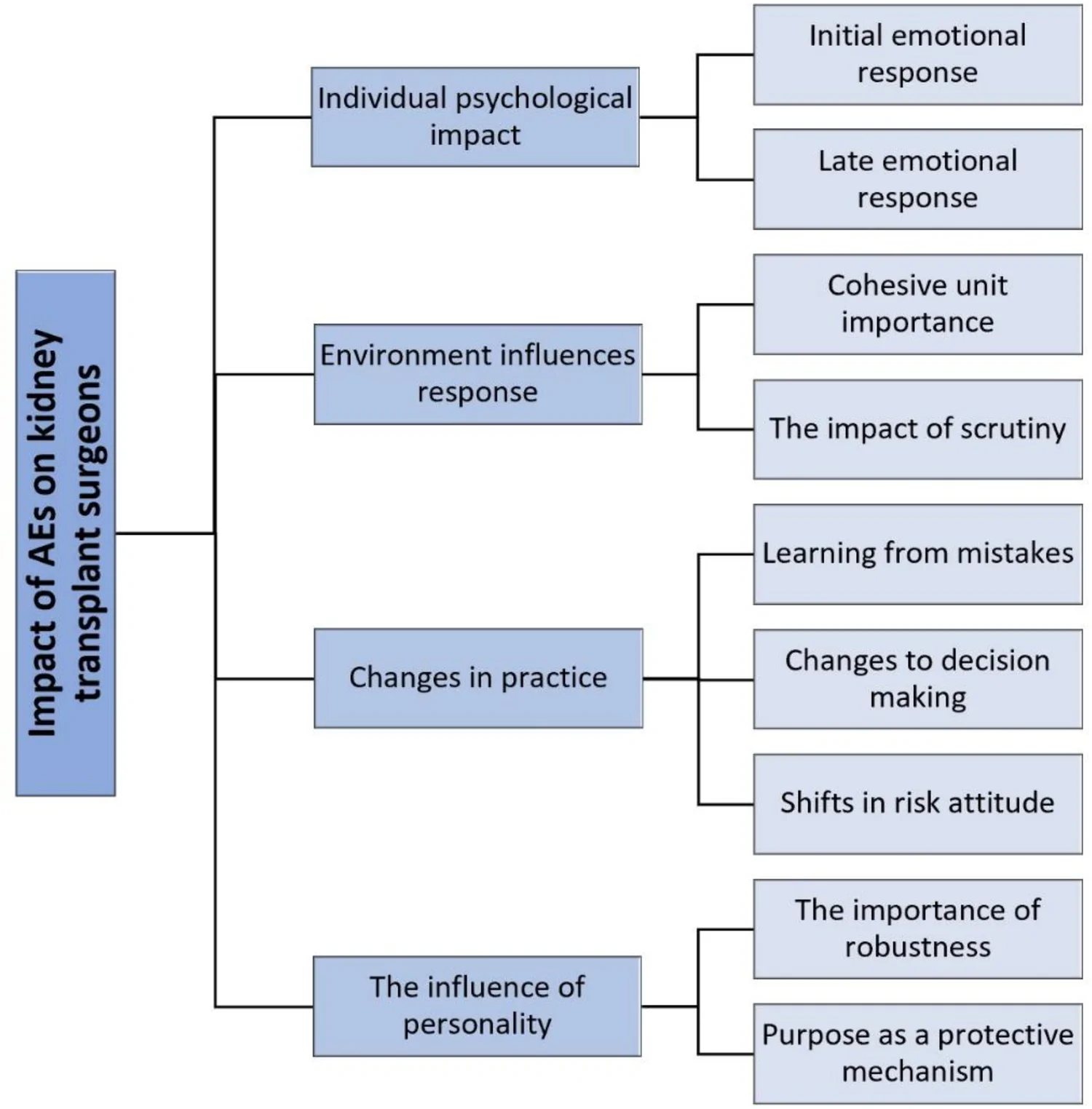
Flow chart depicting themes alongside their subsequent subthemes.

### Theme 1: AEs have an individual psychological impact

#### Initial emotional response

Participants unanimously experienced an initial emotional response following their AE. Surgeons often shared in the trauma of the patient, describing feelings of sadness, anxiety, guilt and frustration. For example: “*devasted, devasted, obviously, really upset”* (Subject 7; Table 1). Some surgeons recounted mentally replaying the event, attempting to understand what had happened.

**Table 1:** Themes and subthemes, with supporting illustrative quotations from interviews.

**Theme 1: AEs have an individual psychological impact**	
**1.1: Initial emotional response**	*“There was obviously immediate sadness. I felt very sorry for him.” - S006*
	*“So, the immediate anxiety that … have I forgotten something in my homework thing.” - S006*
	*“Driving home … I'm thinking about the whole operation, non-stop.” - S008*
	*“Devastated, devastated, obviously, really upset.” - S007*
	*“It was incredibly frightening at the time and a bit traumatic.” - S015*
	*“I was absolutely devastated.” - S016*
**1.2: Late emotional response**	*[“And how'd you feel about the event now?”]* “*Still awful. She’s had 15 years dialysis because of me. If I’d maybe done a better job. If I’d changed things, who knows?” - S007*
	*“You don’t forget about it. And it carries with you. And you still think about it. And every time you see their name, you shudder.” - S007*
	*“There are definitely ones … where I've gone right that was my mistake … and those I have kind of for a long time, harboured the guilt.” - S006*
	*“You still feel bad, but it’s not at the same intensity.” - S013*
	*[“How do you feel about that event now?”]* “*Same as I did then I think which is it was very upsetting.” - S018*
**Theme 2: The surgical environment influences response**	
**2.1: The benefits of a cohesive unit**	*“I've talked about my supportive colleagues, … and there’s definitely a sense that we're doing this as part of a team, not just on your own, that's very good.” -* S006
	*“But certainly the most input that I got was the conversation afterwards with a few people. This is what happened. What do you think? And then chatting about it. That’s definitely the best feedback.” -* S008
	*“Because it didn’t feel like support. It was just organic.” - S004*
	*“I called a colleague in to help me, and it was a bit of a difficult dynamic between the two of us as consultant colleagues … The fact that it came in this case that was just a real challenge with it.” - S010*
	*“I suppose I'm very lucky. I work in a unit where, actually, when something bad happens, everybody goes and talks about it.” - S013*
	*“For me personally, there probably is services, but it wouldn’t be something that would be useful for me.” - S008*
**2.2: The impact of scrutiny following an AE**	*“Not only do you question your own judgement, but my fear is that you've got somebody else in the team who’s also questioning your judgement and questioning your skills.” - S010*
	*“At times, what I've thought about changing my mind is not because I felt it was the wrong thing to do, but it’s more about the perception of your peers … in a heavily scrutinised environment.” - S004*
	*“The anxiety now is driven by the fact that there’s an external body that will take these statistics without knowing the context and process them in a way that can be deleterious to my department and to my colleagues.” - S004*
	*“Just in my mind I was thinking ‘what do they think about me? Do they think I’m competent to do this operation?’ I don’t think they were but I certainly felt that way.” - S017*
	*“It seemed to me to be about looking for blame. There was quite aggressive questioning not just of me but of everybody… I think it was destructive.” - S018*
	*“The M&M meetings are actually quite important, and you have to recognise if you've done something wrong … Why did you do that? What affected you? And how can you stop that? How can everybody else stop doing that? And you can’t be personal about that.” - S007*
**Theme 3: Changes in practice following an AE**	
**3.1: Learning from mistakes**	*“It’s clear at that particular point that I wasn’t very good at it or particularly amazing, but I suppose I'd use that to push to get a bit better.” - S003*
	*“Do not let it go to waste. There'll be a learning opportunity from here … You have to learn from it so you can improve.” - S008*
	*“You have a responsibility to do that. For every patient that comes to harm, you must reflect on it and see if there’s anything you can learn.” - S009*
	*“I have actually gone to see how my colleagues do their arterial anastomoses, because although I thought I had a technique that worked well for me at the time clearly it didn't.” - S003*
**3.2: Changes to decision making**	*“If your decision was right and the outcome was bad, that doesn’t mean you should change your decision next time.” - S006*
	*“Once you have multiple, multiple things and you can’t learn from it, then there’s maybe a deeper issue.” - S008*
	*“I think I have noticed that my an anastomotic time has got slightly longer since this happened and so I think maybe even perhaps subconsciously I am checking a bit more than before.” - S016*
	*“I think it’s impacted my approach to complex transplant counselling, and how I think it’s really a multi-step process” - S004*
**3.3: Shifts in risk attitude**	*“It was a temporary shift definitely to more conservative … just because it jits your confidence” - S008*
	*“Adverse events close to the time invariably impact on your attitude to risk. But I think as time passes, you go back to your steady state of what your risk appetite is, and that’s obviously based on what you feel is the right thing for the patient” - S004*
	*“We do take a lot of risks for our patients, but that’s counteracted by the fact that a life on dialysis is a miserable life with high risk of death as well.” - S010*
	*“The easiest thing to do is to be risk averse … and that to my mind is very bad medicine because patients are not getting the treatment that they deserve.” - S018*
	*“If your first case is a complete disaster, I think that will … have a massive impact on the rest of your career, and will make you much more conservative and much less willing to accept risk.” - S003*
**Theme 4: The influence of personality on responses**	
**4.1: The importance of robustness**	*“And you put an end to it. And I'm pretty good, once I put an end to it - that’s it. I don’t think about it anymore.” - S008*
	*“The lesson from this is actually just reinforced that you have to almost pause your own trauma and process it later.” - S004*
	*“But reflecting back on it … I don’t think I could do anything differently. And I think from a psychological point of view, that’s really helpful.” - S010*
	*“The challenge is to make sure that you remain as a functional surgeon, isn’t it? That’s the importance in some respects, I think recognizing that you have a job to perform as a surgeon is still important, that you need to keep things in perspective and continue to work.” - S012*
	*“But you’ve got to be able to deal with it, and not let it crush you. And I don’t have an answer to how you do that, you’ve got to find works for you, everyone is different.” - S009*
	*“Frankly, your emotions, in my opinion, are in that situation, secondary. You just got to put them away somewhere, compartmentalise them.” - S004*
**4.2: Purpose as a protective mechanism**	*“Is my core purpose to massage figures of our department, or is it to do what the patient would have wanted me to do in their best interests? I've come to the conclusion, if I had not put that kidney in, I would have done it more for departmental statistics rather than for the individual.” - S004*
	*“You're here for a purpose. You're given skills. You're given training and education. There’s somebody actually needing your help that you’re trained to do. That's your job.” - S007*
	*“You have to have faith in your ability as a surgeon and recognize that complications happen, but also to have faith in the process that okay, yeah it is a devastating outcome for this particular patient, but to have faith that what we do generally actually makes a huge difference” - S012*
	*“What is the purpose of my existence? Well, when things go wrong, it’s for me to take responsibility for it.” - S006*

#### Late emotional response

The long-term emotional response was more varied than the initial reaction. Some participants continued to feel as intensely about the event, whereas others have a more subdued reflection now. In both instances, the emotional burden had faded from daily consciousness, but remained accessible, for example when seeing the same patient again or when in a similar clinical scenario. Surgeons described carrying these events with them throughout their careers and learning to live with the burden of their patients’ morbidity.

External factors, such as the reaction of a patient’s family; the severity of the AE; whether the AE included an overtly technical error; and seniority at the time of the event, were identified as influencing the response.

The BFI findings provide descriptive context regarding personality characteristics within this cohort. Mean neuroticism scores were higher among surgeons reporting greater long-term emotional impact than among those reporting lesser impact (3.28 vs. 2.08, Figure 2). DOSPERT scores were comparable across all domains (Supplementary Figure S2). Given the sample size and qualitative basis for group classification, this comparison is descriptive and not associative.

**Figure 2:**
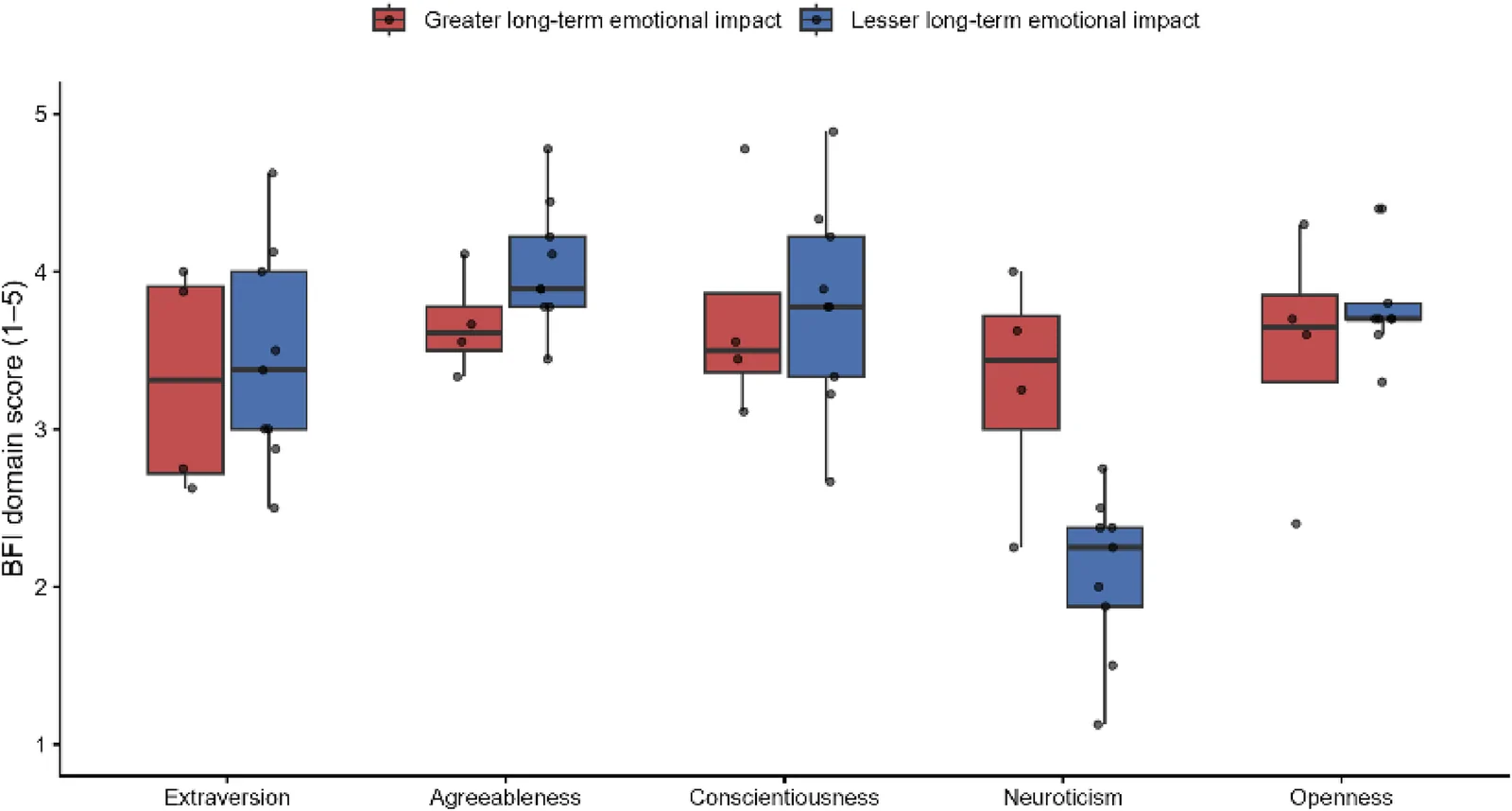
Mean BFI scores for participants per domain, grouped into participants experiencing a greater long-term emotional impact following an AE and a lesser long-term emotional impact. (*n* = 13).

### Theme 2: the surgical environment influences response

#### The benefits of a cohesive unit

Participants consistently emphasised that the presence of a supportive surgical unit was foundational in navigating the emotional and professional aftermath of an AE. Although Units 1 and 2 contributed the largest number of participants, the importance of peer support was described across all units, rather than being specific to any individual centre. Informal support provided by colleagues was widely regarded as the most effective coping mechanism. Colleagues were able to provide perspective and reassurance and served as a reminder that AEs are a shared, common experience within transplantation. This relational support was often viewed as inherent team culture rather than support and preferred over accessing formal support services. Conversely, a fragmented team dynamic was found to add to the burden of an AE.

#### The impact of scrutiny following an AE

Scrutiny was reported to evoke feelings of doubt in surgical capabilities, with some surgeons stating that decision-making processes became governed by concern over peer perceptions rather than clinical reasoning. For example: “*At times, what I've thought about changing my mind is not because I felt it was the wrong thing to do, but it's more about the perception of your peers … in a heavily scrutinised environment”* (Subject 4; Table 1). Pressure from external bodies on departmental metrics were also found to add to the consequences of an AE. Scrutiny from colleagues was often a perceived pressure – shaped more by internalised assumptions about peers’ judgements than by explicit criticism. In contrast, scrutiny from audits and external reviews was felt to be more direct and tangible.

Despite this, a positive aspect to scrutiny was found, particularly within the morbidity and mortality (M&M) process. Some stated that M&M was important from a learning and accountability perspective, ensuring that errors were thoroughly reviewed and understood, thus facilitating the coping process.

### Theme 3: changes in practice following an AE

#### Learning from mistakes

Surgeons commonly expressed a desire to improve following an AE. This was driven by surgical pride and motivation to perform to the best of their abilities. There was a collective feeling that an AE should be used as a learning experience. Critical self-assessment was required to enable improvement following an AE. This was supported by a communal element, with reliance on the advice and knowledge of colleagues.

#### Changes to decision-making

Participants reported changes to their practice should be based on their decision making and not solely outcomes. A balance between making reactive changes and maintaining confidence in their pre-existing skills was explored. Participants were conscious of the importance of not overcorrecting based on isolated incidents yet still being able to recognise patterns that could indicate systemic issues necessitating changes to their perioperative decision-making. Some surgeons reported changing their practice, especially how they counselled future patients after an AE, with greater formalisation and risk explanation in the consent process.

#### Shifts in risk attitude

Surgeons appreciated differences in baseline risk tolerance between themselves and colleagues, but many noted a natural shift to being more conservative in the immediate aftermath of an AE. Eventually, there is a recalibration of risk appetite based on surgical confidence and patient needs. Multiple variables were found to influence surgeons’ risk calculus, including personality, performance metrics and experience. Ultimately most believed that it was determined by the comparison of risk between current quality of life and the intervention.

### Theme 4: the influence of personality on responses

Mean BFI domain scores were compared with published scores from other surgical subspecialties (30, 31) to generate a contextual profile (Figure 3). Participants consistently believed there to be a link between personality and AE responses.

**Figure 3:**
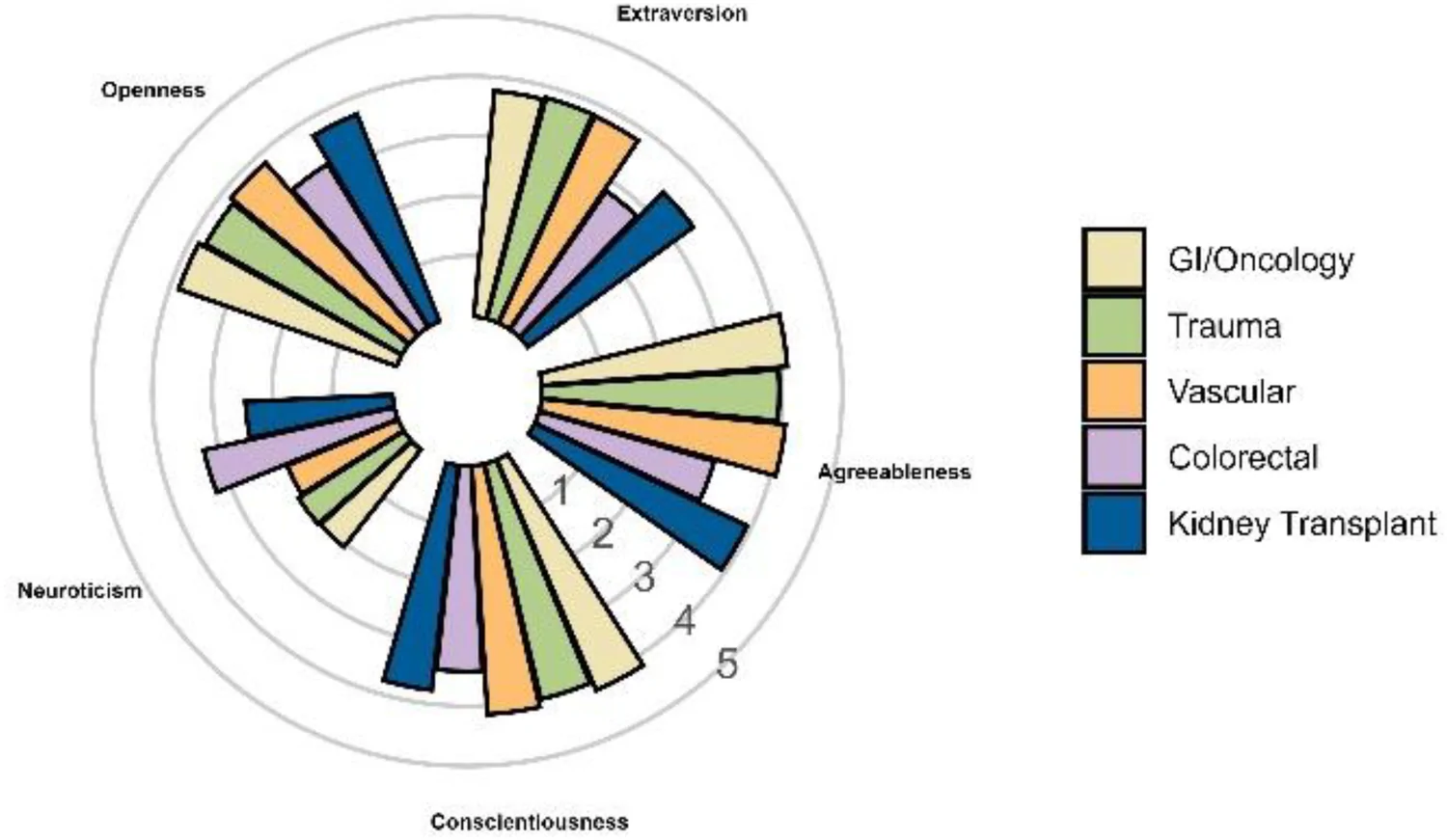
Mean domain scores per surgical subspecialty group. Mean BFI scores per domain for our participants in comparison to other surgical subspecialties from relevant studies (24, 25).

#### The importance of robustness

Participants invariably described a degree of “robustness” required to be able to cope with the repercussions of an AE. Maintenance of emotional wellbeing and professional functioning was frequently credited to the ability to “*draw a line”* following an AE. Participants often alluded to a need to temporarily pause their own emotional response so they could continue their clinical tasks, addressing this at a later stage.

#### Purpose as a protective mechanism

Surgeons described a period of introspection and reflection on their purpose after an AE. This helped reinforce the fundamental values of a career in transplant surgery: a duty to patients, to shoulder responsibility and perform to the standards that their training has allowed. Circling back to this core purpose served as a grounding and coping mechanism. Notably, this didn't disregard the impact of the AE, rather reframed it as part of the job's inherent challenge. For example: “*You're here for a purpose. You're given skills. You're given training and education. There's somebody actually needing your help that you’re trained to do. That's your job.”* (Subject 7; Table 1).

## Conclusion

Our study reports the impact of peri-operative adverse events on the psychosocial wellbeing of kidney transplant surgeons. In recent years there has been growing awareness of the emotional impact of adverse outcomes in surgery (8, 9, 32–35); however to our knowledge this has never been studied within transplantation before.

Akin to studies in other surgical fields (9, 14–18, 34, 36), transplant surgeons reported immediate sadness and guilt following an AE. The Bowlby and Parkes model (37) provides a framework by which the emotional response to AEs may be better understood. Figure 4 attributes selected responses from interviews into the four-stage model and supports the concept of SVS (4), with all surgeons describing an acute reaction, while the longer-term emotional impact is more varied.

**Figure 4:**
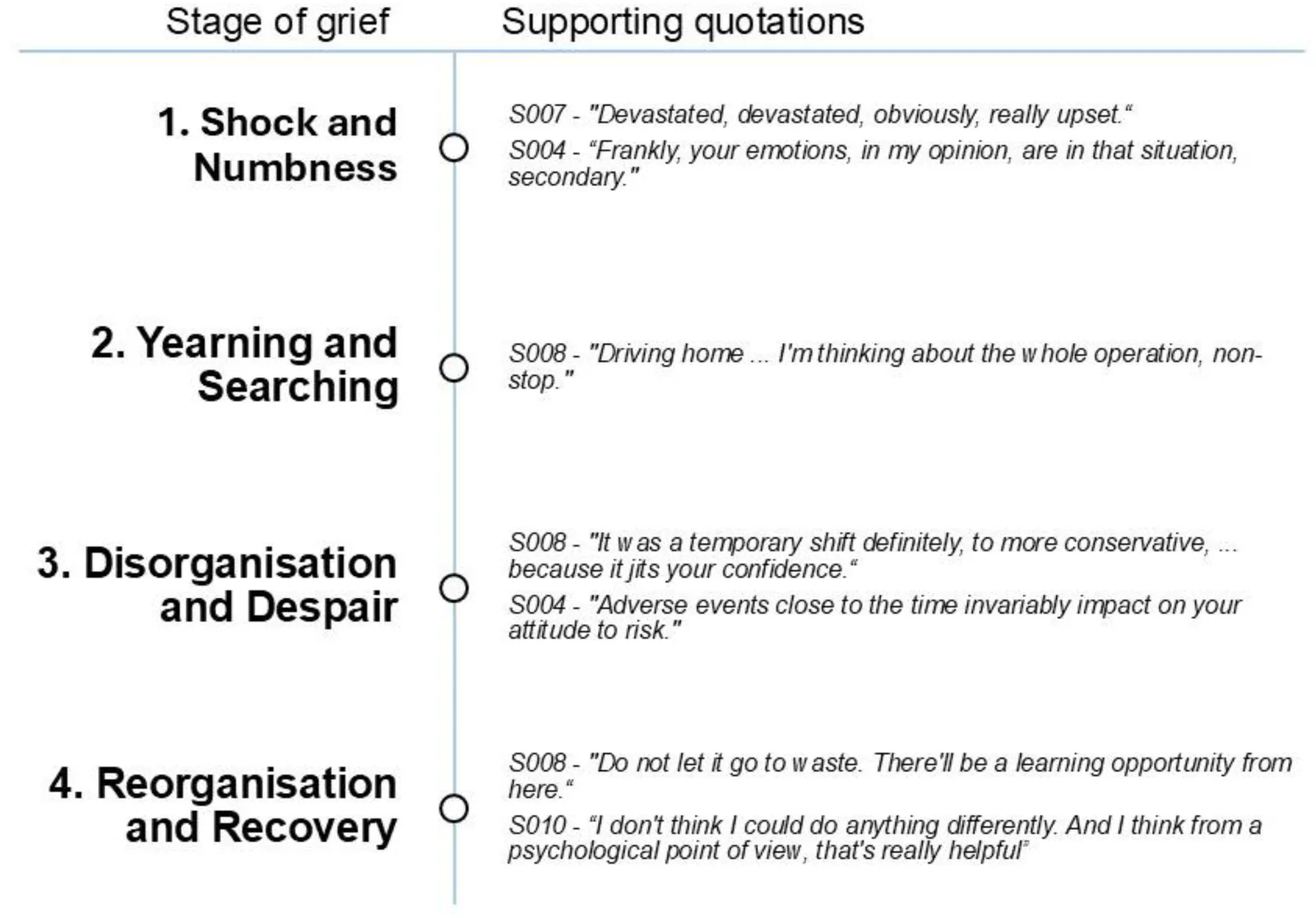
Illustrative mapping of selected interview quotations onto Bowlby and Parkes’ four-stage grief model (37).

As in other surgical subspecialties (14, 17, 38), more severe AEs were observed to have a greater lasting emotional impact on kidney transplant surgeons. Similarly, if the AE was considered a technical error, this was associated with a greater emotional response. Participants reported an inevitability of AEs within surgery, possibly explaining why a non-technical error is experienced less harshly than a technical error (18, 34, 39). Seniority at the time of the event has been shown to influence how AEs are dealt with. Reduced clinical experience was perceived to be associated with increased vulnerability in surgical confidence (17, 18). Interestingly, some participants also described that being one of the most experienced surgeons in a department can be difficult to manage. In this scenario, there is an expectation of emotional resilience and a perceived lack of peer reassurance. Our data also suggest that AEs may have a greater emotional burden if accompanied by antagonistic responses from the patients’ families. Confronting hostility, whilst still processing the AE can amplify the emotional response (34).

Surgeons required both external and internal processing of grief and guilt after an AE. External validation could be provided by colleagues, either senior surgeons sharing similar past experiences or immediate peers demonstrating that such events are not unique (35, 40). However, participants also described the need for internal acceptance of the event, allowing them to move beyond the case and continue to perform at the level needed by patients. Acceptance of the event was often aided by M&M meetings. Responses highlighted that a well-conducted M&M process allowed the surgeon to learn from any mistake and take responsibility, in a manner that “you can't be personal” about. Participants distinguished this constructive scrutiny from perceived colleague scrutiny, which was described as harmful to practice and associated with feelings of self-doubt. This reinforces the importance of a supportive peer environment (12).

Although some participants suggested that more timely support processes would be helpful in dealing with AEs, existing literature has highlighted limited effectiveness of single-session psychological debriefing following traumatic events (41–44). Furthermore, aspects of surgical culture may act as a barrier to surgeon engagement in formal support structures, with surgeons expected to have the capacity to deal with the events alone (45–47). This serves to strengthen our finding that the most effective support mechanism came, not from structured interventions, but collegial relationships within a cohesive team (12, 32). An environment where staff are comfortable in admitting mistakes and learning from them is beneficial to patient outcomes. This psychological safety is shaped by continuous interpersonal interactions amongst co-workers (48). There is evidence of the efficacy of Schwartz rounds, which serve to improve team cohesion, support staff wellbeing and encourage compassionate reflection (49–51). The potential benefits of this lie in fostering peer support and emotional resilience rather than problem-solving. Such services could be particularly beneficial in the complex, multidisciplinary transplant setting.

Our results also demonstrate the role that personality and professional identity have in dealing with AEs. “Purpose” as a transplant surgeon proved to be an effective protective mechanism, with existing evidence clarifying that this predicts a better emotional recovery from negative stimuli (52). Our results suggest resilience (perseverance in the face of adversity) is emotionally protective following a complication, allowing surgeons to sustain practice despite emotional challenges (53, 54). Both innate characteristics and environmental factors like support systems and workplace culture play a role in acquisition of resilience (53, 55) and it often co-exists with the ability for growth and improvement following an AE. Given the emotionally protective role that resilience plays, our findings show that it could be a potential target for structured intervention. Recent Enhanced Stress Resilience Training (ESRT) in Core Surgical Training showed improvements in burnout, stress and mindfulness in participants that fully engaged with the 5-week course (56). Lebares et al. reported the feasibility of such interventions, with no detrimental effect on surgical training (57, 58). Whether similar resilience-focused training could benefit transplant surgeons warrants further investigation.

While previous studies have shown poor outcomes alter decision-making (12, 16), our results suggest that changes in practice were driven primarily by the perception that the adverse outcome resulted from poor-decision making, rather than the technical outcome itself. Participants acknowledged transplant surgery is inherently high risk, but the reality of not performing a transplant – and remaining on dialysis –whilst perhaps safer for the surgeon, - also carries substantial risk for the patient (59). Kidney transplantation requires constantly balancing the competing risks of action versus inaction. Participants considered that avoiding operating because of personal risk perception may be as paternalistic as proceeding based solely on a belief in its benefit. Given that poor outcomes are an inescapable part of transplant practice, focus is shifted towards decision-making and being able to recalibrate risk following an AE.

Our study is limited in its size and scope. Whilst participants covered a range of centres across the UK, the majority were based across two centres. Consequently, exploration of collegial matters was similar in these interviews. Although this provided rich insight into the benefits of a cohesive unit, it meant that there was also a lack of information on the harms of a fragmented team dynamic and opinions on how this could be improved (34). Furthermore, the interviews being conducted by a medical student alone introduces a potential power dynamic and knowledge gap.

The limited sample size meant that our quantitative results could only be used to provide contextual depth, and not to find correlations with qualitative data, highlighting the need for more extensive personality assessment in this cohort in the future.

In summary, this mixed-methods study explored the impact of perioperative AEs on kidney transplant surgeons, demonstrating the significant personal and professional consequences of these events. The findings provide novel insight into the detrimental psychological effects of complications, and how the unique responsibilities of the job impact risk attitude and decision-making following an AE. Professional identity was identified as a protective mechanism for participants, and that both internal and external coping strategies were necessary following an AE. These findings highlight the potential value of strengthening collegial support within transplant units, and further evaluating formal support systems, which remain underused. Further multicentre research is required to confirm these findings and to evaluate potential approaches to supporting surgeons following AEs.

## Data Availability

The raw data supporting the conclusions of this article will be made available by the authors, without undue reservation.

## References

[1] Skelly CL Cassagnol M Munakomi S. Adverse events. In: Munakomi S, editor. Advanced Practice and Leadership in Radiology Nursing (2023). p. 213–21. 10.1007/978-3-030-32679-1_19 PubMed PMID: 32644389.

[2] Mittlmeier A Canal C Dell-Kuster S Pape HC Neuhaus V. Association between documented intraoperative and postoperative adverse events: an analysis of over 100,000 surgical trauma cases. Br J Surg. (2024) 111(Supplement_3):21. 10.1093/BJS/ZNAE118.066

[3] Anderson O Davis R Hanna GB Vincent CA. Surgical adverse events: a systematic review. Am J Surg. (2013) 206(2):253–62. 10.1016/J.AMJSURG.2012.11.009 PubMed PMID: 23642651. 23642651

[4] Wu AW. Medical error: the second victim. Br Med J. (2000) 320(7237):726–7. 10.1136/BMJ.320.7237.726 PubMed PMID: 10720336. PMC1117748 10720336

[5] Marmon LM Heiss K. Improving surgeon wellness: the second victim syndrome and quality of care. Semin Pediatr Surg. (2015) 24(6):315–8. 10.1053/J.SEMPEDSURG.2015.08.011 PubMed PMID: 26653167. 26653167

[6] Han K Bohnen JD Peponis T Martinez M Nandan A Yeh DD. The surgeon as the second victim? Results of the Boston intraoperative adverse events surgeons’ attitude (BISA) study. J Am Coll Surg. (2017) 224(6):1048–56. 10.1016/J.JAMCOLLSURG.2016.12.039 PubMed PMID: 28093300. 28093300

[7] Waterman AD Garbutt J Hazel E Dunagan WC Levinson W Fraser VJ. The emotional impact of medical errors on practicing physicians in the United States and Canada. Jt Comm J Qual Patient Saf. (2007) 33(8):467–76. 10.1016/S1553-7250(07)33050-X PubMed PMID: 17724943. 17724943

[8] Bryan J Ketley A Cavanagh K Bisset C Moug S Wyld L. Second victim syndrome in surgeons: systematic review and meta-analysis of the impact of adverse events on surgeons. Br J Surg. (2025) 113(1):258. 10.1093/BJS/ZNAF258 PubMed PMID: 41499327. PMC12777971 41499327

[9] Awuah WA Aderinto N Ahluwalia A Poornaselvan J Tan JK Bharadwaj HR. Beyond the operating room: addressing the “second-victim” phenomenon in surgical practice. Eur J Med Res. (2024) 29(1):486. 10.1186/S40001-024-02084-Z PubMed PMID: 39367498. 39367498 PMC11452961

[10] Le HD Wolinska JM Baertschiger RM Himidan SA. Complication is inevitable, but suffering is optional—psychological aspects of dealing with complications in surgery. Eur J Pediatr Surg. (2023) 33(3):181–90. 10.1055/S-0043-1767830 PubMed PMID: 36948212. 36948212 PMC10188232

[11] Rivard SJ Vitous CA De Roo AC Bamdad MC Jafri SM Byrnes ME. The captain of the ship.” A qualitative investigation of surgeon identity formation. Am J Surg. (2022) 224(1):284–91. 10.1016/J.AMJSURG.2022.01.010 PubMed PMID: 35168761. 35168761 PMC9531326

[12] Bisset CN Moug SJ Oliphant R Dames N Parson S Cleland J. Influencing factors in surgical decision-making: a qualitative analysis of colorectal surgeons’ experiences of postoperative complications. Colorectal Dis. (2024) 26(5):987–93. 10.1111/CODI.16943 PubMed PMID: 38485203. 38485203

[13] Bisset CN Moug SJ Oliphant R Dames N Cleland J. Surgeon perceptions of personality as an influencing factor on anastomotic decision-making: a qualitative analysis. Colorectal Dis. (2024) 26(8):1608–16. 10.1111/CODI.17078 PubMed PMID: 39162024. 39162024

[14] Drudi LM D’Oria M Bath J Van Nispen J Smeds MR. Postoperative complications and their association with post-traumatic stress disorder in academic vascular surgeons. Journal of Vascular Surgery. (2023) 77:899–905.e1. 10.1016/j.jvs.2022.10.056 PubMed PMID: 36402248. 36402248

[15] Lin JS Olutoye OO Samora JB. To err is human, but what happens when surgeons err? J Pediatr Surg. (2023) 58(3):496–502. 10.1016/J.JPEDSURG.2022.06.019 PubMed PMID: 35914964. 35914964

[16] Akyol C Celik SU Koc MA Bayindir DS Gocer MA Karakurt B. The impact of patient deaths on general surgeons’ psychosocial well-being and surgical practices. Front Surg. (2022) 9:898274. 10.3389/FSURG.2022.898274 PubMed PMID: 35574543. 35574543 PMC9096651

[17] Collings R Potter C Gebski V Janda M Obermair A. The impact of surgical complications on obstetricians’ and gynecologists’ well-being and coping mechanisms as second victims. Am J Obstet Gynecol. (2025) 232(1):104.e1–104.e12. 10.1016/j.ajog.2024.07.043 PubMed PMID: 39111518. 39111518

[18] Turner K Bolderston H Thomas K Greville-Harris M Withers C McDougall S. Impact of adverse events on surgeons. Br J Surg. (2022) 109(4):308–10. 10.1093/BJS/ZNAB447 PubMed PMID: 35084452. 35084452

[19] Shanafelt TD Balch CM Bechamps G Russell T Dyrbye L Satele D. Burnout and medical errors among American surgeons. Ann Surg. (2010) 251(6):995–1000. 10.1097/SLA.0B013E3181BFDAB3 PubMed PMID: 19934755. 19934755

[20] American Medical Association. Guidelines for Organ Transplantation from Deceased Donors | AMA-Code [Internet]. Available online at: https://code-medical-ethics.ama-assn.org/ethics-opinions/guidelines-organ-transplantation-deceased-donors (Accessed 2025 April 22)

[21] NHS Blood and Transplant. Annual report on kidney transplantation [Internet]. (2024). Available online at: https://nhsbtdbe.blob.core.windows.net/umbraco-assets-corp/34295/nhsbt-kidney-transplantation-report-2324.pdf (Accessed 2025 April 18)

[22] Kassam AF Axelrod DA Geevarghese SK. Moral injury: an unspoken burden of transplant surgery. Am J Transplant. (2025) 25(1):32–7. 10.1016/J.AJT.2024.08.012 PubMed PMID: 39159721. 39159721

[23] Tong A Sainsbury P Craig J. Consolidated criteria for reporting qualitative research (COREQ): a 32-item checklist for interviews and focus groups. Int J Qual Health Care. (2007) 19(6):349–57. 10.1093/INTQHC/MZM042 PubMed PMID: 17872937. 17872937

[24] Santos G Jones MW. Prevention of surgical errors. StatPearls. (2023). PubMed PMID: 37276278. 37276278

[25] Giddins G. Surgical complications: errors and adverse events. J Hand Surg Eur Vol. (2024) 49(2):142–8. 10.1177/17531934231206317 38315132

[26] Goldberg LR. The structure of phenotypic personality traits. Am. Psychol. (1993) 48(1):26–34. 10.1037/0003-066X.48.1.26 8427480

[27] John OP Srivastava S. The big-five trait taxonomy: history, measurement, and theoretical perspectives. In: Pervin LA John OP, editors. Handbook of Personality: Theory and Research. New York: Guilford Press (1999). p. 102–38.

[28] Blais AR Weber EU. A domain-specific risk-taking (DOSPERT) scale for adult populations. Judgm Decis Mak. (2006) 1(1):33–47. 10.1017/s1930297500000334

[29] Braun V Clarke V. One size fits all? What counts as quality practice in (reflexive) thematic analysis? Qual Res Psychol. (2021) 18(3):328–52. 10.1080/14780887.2020.1769238

[30] Sier VQ Schmitz RF Wertenbroek RWAM Schepers A Van Der Vorst JR Beetz I. Surgeon personality diversity across generations and subspecialties. Surgeon. (2025) 23(2):78–86. 10.1016/j.surge.2025.02.003 PubMed PMID: 39966018. 39966018

[31] Bisset CN Ferguson E MacDermid E Stein SL Yassin N Dames N. Exploring variation in surgical practice: does surgeon personality influence anastomotic decision-making? Br J Surg. (2002) 109(11):1156–63. 10.1093/bjs/znac200 PMC10364753 35851801

[32] O’Meara S Doherty E Walsh K. Where do we go from here? The personal impact of adverse events and complications in surgeons: results from a cross-sectional study. Surgeon. (2025) 23(2):73–7. 10.1016/J.SURGE.2025.01.001 39809697

[33] Serou N Sahota L Husband AK Forrest SP Moorthy K Vincent C. Systematic review of psychological, emotional and behavioural impacts of surgical incidents on operating theatre staff. BJS Open. (2017) 1(4):106–13. 10.1002/BJS5.21 29951612 PMC5989958

[34] Pinto A Faiz O Bicknell C Vincent C. Surgical complications and their implications for surgeons’ well-being. Br J Surg. (2013) 100(13):1748–55. 10.1002/BJS.9308 24227360

[35] Choi SHJ Yan TD Misskey J Chen JC. The emotional impact and coping mechanisms following adverse patient events among Canadian vascular surgeons and trainees. Vasc Endovascular Surg. (2024) 58(3):294–301. 10.1177/15385744231209914 37878392

[36] Patel AM Ingalls NK Mansour MA Sherman S Davis AT Chung MH. Collateral damage: the effect of patient complications on the surgeon’s psyche. Surgery. (2010) 148(4):824–30. 10.1016/J.SURG.2010.07.024 PubMed PMID: 20727563. 20727563

[37] Bowlby J Parkes C. Separation and loss within the family. In: The Child in His Family: International Yearbook of Child Psychiatry and Allied Professions. (1970). Vol. 1:197–216 p.

[38] Chong RIH Yaow CYL Chong NZ-Y Yap NLX Hong ASY Ng QX. Scoping review of the second victim syndrome among surgeons: understanding the impact, responses, and support systems. Am J Surg. (2024) 229:5–14. 10.1016/j.amjsurg.2023.09.045 PubMed PMID: 37838505. 37838505

[39] Vitous CA Byrnes ME De Roo A Jafri SM Suwanabol PA. Exploring emotional responses after postoperative complications: a qualitative study of practicing surgeons. Ann Surg. (2022) 275(1):E124–31. 10.1097/SLA.0000000000004041 PubMed PMID: 33443904. 33443904 PMC9437841

[40] El Hechi MW Bohnen JD Westfal M Han K Cauley C Wright C. Design and impact of a novel surgery-specific second victim peer support program. J Am Coll Surg. (2020) 230(6):926–33. 10.1016/J.JAMCOLLSURG.2019.10.015 PubMed PMID: 31857209. 31857209

[41] Litz BT Gray MJ Bryant RA Adler AB. Early intervention for trauma: current status and future directions. Clin Psychol Sci Pract. (2002) 9(2):112–34. 10.1093/CLIPSY.9.2.112

[42] Bisson JI Jenkins PL Alexander J Bannister C. Randomised controlled trial of psychological debriefing for victims of acute burn trauma. Br J Psychiatry. (1997) 171(1):78–81. 10.1192/BJP.171.1.78 PubMed PMID: 9328501. 9328501

[43] Van Emmerik AAP Kamphuis JH Hulsbosch AM Emmelkamp PMG. Single session debriefing after psychological trauma: a meta-analysis. Lancet. (2002) 360(9335):766–71. 10.1016/S0140-6736(02)09897-5 PubMed PMID: 12241834. 12241834

[44] Sijbrandij M Olff M Reitsma JB Carlier IVE Gersons BPR. Emotional or educational debriefing after psychological trauma: randomised controlled trial. Br J Psychiatry. (2006) 189(2):150–5. 10.1192/BJP.BP.105.021121 PubMed PMID: 16880485. 16880485

[45] Supporting surgeons after adverse events — Royal College of Surgeons [Internet]. Available online at: https://www.rcseng.ac.uk/standards-and-research/standards-and-guidance/good-practice-guides/supporting-surgeons-after-adverse-events/guide/#refs [Accessed 2025 April 14]

[46] Balch CM Shanafelt TS. Dynamic tension between success in a surgical career and personal wellness: how can we succeed in a stressful environment and a “culture of bravado”? Ann Surg Oncol. (2011) 18(5):1213–6. 10.1245/S10434-011-1629-Z/METRICS 21369737

[47] Biggs S Waggett HB Shabbir J. Impact of surgical complications on the operating surgeon. Colorectal Dis. (2020) 22(9):1169–74. 10.1111/codi.15021 32065472

[48] Associate ACE Managing the risk of learning: Psychological safety in work teams [Internet]. (2002). Available online at: https://www.hbs.edu/ris/Publication%20Files/02-062_0b5726a8-443d-4629-9e75-736679b870fc.pdf [Accessed 2025 April 19]

[49] Robert G Philippou J Leamy M Reynolds E Ross S Bennett L. Exploring the adoption of schwartz center rounds as an organisational innovation to improve staff well-being in England, 2009–2015. BMJ Open. (2017) 7(1):e014326. 10.1136/BMJOPEN-2016-014326 PubMed PMID: 28057662. 28057662 PMC5223680

[50] Taylor C Xyrichis A Leamy MC Reynolds E Maben J. Can schwartz center rounds support healthcare staff with emotional challenges at work, and how do they compare with other interventions aimed at providing similar support? A systematic review and scoping reviews. BMJ Open. (2018) 8(10):e024254. 10.1136/BMJOPEN-2018-024254 PubMed PMID: 30341142. 30341142 PMC6196967

[51] Pepper JR Jaggar SI Mason MJ Finney SJ Dusmet M. Schwartz rounds: reviving compassion in modern healthcare. J R Soc Med. (2012) 105(3):94–5. 10.1258/JRSM.2011.110231 PubMed PMID: 22434804. 22434804 PMC3308642

[52] Schaefer SM Morozink Boylan J Van Reekum CM Lapate RC Norris CJ Ryff CD. Purpose in life predicts better emotional recovery from negative stimuli. PLoS One. (2013) 8(11):e80329. 10.1371/JOURNAL.PONE.0080329 PubMed PMID: 24236176. 24236176 PMC3827458

[53] Zwack J Schweitzer J. If every fifth physician is affected by burnout, what about the other four? Resilience strategies of experienced physicians. Acad Med. (2013) 88(3):382–9. 10.1097/ACM.0B013E318281696B PubMed PMID: 23348093. 23348093

[54] Epstein RM Krasner MS. Physician resilience: what it means, why it matters, and how to promote it. Acad Med. (2013) 88(3):301–3. 10.1097/ACM.0B013E318280CFF0 23442430

[55] Chbeir S Carrión V. Resilience by design: how nature, nurture, environment, and microbiome mitigate stress and allostatic load. World J Psychiatry. (2023) 13(5):144–59. 10.5498/WJP.V13.I5.144 PubMed PMID: 37303926. 37303926 PMC10251360

[56] Luton OW James OP Mellor K Eley C Hopkins L Robinson DBT. Enhanced stress resilience training for UK surgical trainees. Effect and Evolution Evaluated. J Surg Educ. (2023) 80(10):1395–402. 10.1016/J.JSURG.2023.07.017 PubMed PMID: 37567800. 37567800

[57] Lebares CC Hershberger AO Guvva EV Desai A Mitchell J Shen W. Feasibility of formal mindfulness-based stress-resilience training among surgery interns: a randomized clinical trial. JAMA Surg. (2018) 153(10):e182734. 10.1001/JAMASURG.2018.2734 PubMed PMID: 30167655. 30167655 PMC6233792

[58] Lebares CC Coaston TN Delucchi KL Guvva EV Shen WT Staffaroni AM. Enhanced stress resilience training in surgeons: iterative adaptation and biopsychosocial effects in 2 small randomized trials. Ann Surg. (2021) 273(3):424–32. 10.1097/SLA.0000000000004145 PubMed PMID: 32773637. 32773637 PMC7863698

[59] Chander S Luhana S Sadarat FNU Parkash O Rahaman Z Wang HY. Mortality and mode of dialysis: meta-analysis and systematic review. BMC Nephrol. (2024) 25(1):1. 10.1186/S12882-023-03435-4 PubMed PMID: 38172835. 38172835 PMC10763097

