# Supplementary material for: The impact of perioperative adverse events on kidney transplant surgeons: a mixed-methods study
Source: Front Transplant. 2026 Oct 14;5:1959604. doi: 10.3389/frtra.2026.1959604 (PMC13713721; doi:10.3389/frtra.2026.1959604)
Supplement: Supplementary file 1 [file Table1.docx]

**Supplementary Appendix**

Topic guide

TOPIC GUIDE: interviews for perioperative adverse events effect on the surgeon

This is a semi-structured topic guide designed to guide the interview. It will be adapted within the context of the participant. The participant is welcome to raise topics that fall outside of the themes explored in the topic guide if they are important to them and their experiences.

**Setting the Scene: *we would like to begin by learning some background knowledge on your career and about the context of the event.***

- Can you tell me a little about your background in transplant surgery
  - years of experience/as a consultant
  - primary role and types of transplants performed
- Can you tell me about your adverse event.
  - Who was the patient, what treatment had they had at this stage, how fit were they for transplant?
  - When did the event take place?
  - Tell me about the kidney
    - What was the context of the offer
    - What donor factors had to be considered
    - How well matched was the kidney to the patient
    - What was the quality of the vascular access
    - What was the recipient’s health
    - Was this a good offer for the patient
  - Were there any concerning factors pre-surgery?
  - What were the long-term consequences for the patient?
  - How do you feel about the event now?
    - Do you think there is anything you could have done personally that would have changed the outcome?

**Emotional Impact: *we are interested in the emotional response that surgeons experience when an adverse event occurs.***

- How did you feel about the event: Anxiety? Stress? Guilt? Anger?
  - At the time
  - In the coming weeks/months
- Did the event affect you emotionally or psychologically over the long term? How?
- To what extent was this event something you felt personally responsible for, in comparison to a wider system responsibility? Why?

**Physical and wider Impact: *we would like to know if the impact of the adverse event has more than an emotional impact, and if it has physical manifestations or wider implications for the surgeons.***

- Did you experience any physical symptoms because of how you were feeling after the event?
  - Sleep problems
  - Energy levels
- How did the event impact your personal life outside of work?
- Did the event affect any of your relationships?
  - Partner
  - Colleagues
  - Family

**Professional Impact: *we are interested in knowing the professional implications of an adverse event on the surgeon.***

- Has this event impacted your clinical practice? If so, how?
- Has this event affected your decision making? If so, how?
- Has this event made you feel under pressure or scrutinised?
  - From where?
  - How has this made you feel?
- Have you asked what other colleagues think or feel about the event?
  - How do you feel about that?

**Support received, coping mechanisms and communications: *this study hopes to analyse the current support systems available, how effective they are, and if there any suggestions that you have yourself about how they can be improved.***

- What strategies did you use to cope with the aftermath of the event?
- What support did you receive following the event? Do you think it was sufficient?
- Who did you go to for support?
- Did you go to your colleagues for support?
  - How did they support you?
  - How did this support evolve or change over time?
  - How did your colleague’s response make you feel?
  - Has is affected your interactions with colleagues or the wider healthcare team?
- Do you think that you were prepared to deal with the event that happened?
- What coping strategies do you think were helpful? Do you think you used any that were less helpful?
- Are there any pre-existing factors that influenced how you dealt with this event?
  - Professional
  - Outside of medicine
  - Do you think that personality is one of them?
- Are there any changes to the support system that you would like to see following your experience?
- What communication have you had with the patient and their family since the event?
  - How did you find this?
- What about future communication with this family/other families?
  - Will the adverse event affect it? In what ways?

**Decision Regret and Risk-Taking behaviour: *we would like to explore the relationship between risk-taking behaviour and adverse events occuring.***

- Do you regret any of the decisions that you made during this surgery?
- If faced with the same situation again, would you do the same thing?
- How does your attitude to risk in surgery compare to your attitude to risk in day-to-day life?
- What influences your decision making and tolerance of risk in transplant surgery?
  - How does this compare to your colleagues?
  - Do you think that personality is an influence? If so, how?
- Has this event changed your attitude to risk as a surgeon? If so, how?
- Has this event impacted your approach to organ acceptance?
  - Are you more risk averse now?
  - Has it affected your attitude towards high-risk recipients and high-risk donor offers?

**Closing Ideas: to end things today we have a couple broader points that we would like to speak about.**

- What is your opinion on how adverse events impact a surgeon’s career
- Do you have any other points you would like to raise based on what we have spoken about today?

DOSPERT scale questionnaire

Supplementary Figure 1: CONSORT diagram outlining participant selection for mixed-methods analysis

Supplementary Figure 2: Mean DOSPERT scores for participants per domain, grouped into participants experiencing a greater long-term emotional impact following an AE and a lesser long-term emotional impact (n=13).

| **Participant characteristics** | **No. of surgeons (N=15)** |
| --- | --- |
| **Gender** |  |
| Male | 11 |
| Female | 4 |
| **Experience as Consultant at Time of Event** |  |
| Early Career (< 5 yrs) | 8 |
| Established (≥ 5yrs) | 7 |
| **Experience as Consultant at Time of Interview** |  |
| Early Career (< 5 yrs) | 6 |
| Established (≥ 5yrs) | 9 |
| **Hospital Location** |  |
| Unit 1 | 5 |
| Unit 2 | 4 |
| Unit 3 | 2 |
| Unit 4, 5, 6 and 7 | 1 |
| **Nature of AE** |  |
| Death following transplant complications | 5 |
| Haemorrhage leading to nephrectomy | 3 |
| Intraoperative haemorrhage | 2 |
| Lack of perfusion post-transplant leading to nephrectomy | 2 |
| Lack of perfusion during transplant | 1 |
| Postoperative wound complication | 1 |
| Severe limb ischaemia secondary to transplant complication | 1 |

Supplementary table 1: Participant demographic table
